# Identification of Universally Applicable and Species-Specific Marker Peptides for *Bacillus anthracis*

**DOI:** 10.3390/life12101549

**Published:** 2022-10-06

**Authors:** Natalie Witt, Domenico Galante, Sandro Andreotti, Mostafa Abdel Glil, Antonio Fasanella, David Meierhofer, Herbert Tomaso

**Affiliations:** 1 Mass Spectrometry Facility, Max Planck Institute for Molecular Genetics, Ihnestraße 63-73, 14195 Berlin, Germany; 2 Bioanalytics, Institute of Biotechnology, Technische Universität Berlin, 13355 Berlin, Germany; 3 Istituto Zooprofilattico Sperimentale della Puglia e della Basilicata, Anthrax Reference Institute of Italy, Via Manfredonia 20, 71121 Foggia, Italy; 4 Bioinformatics Solution Center, Department of Mathematics and Computer Science, Freie Universität Berlin, 14195 Berlin, Germany; 5 Friedrich-Loeffler-Institut, Institute of Bacterial Infections and Zoonoses, Naumburger Str. 96 a, 07743 Jena, Germany

**Keywords:** *Bacillus anthracis*, anthrax, high-resolution mass spectrometry, species-specific marker peptides

## Abstract

Anthrax is a zoonotic infection caused by the bacterium *Bacillus anthracis* (BA). Specific identification of this pathogen often relies on targeting genes located on two extrachromosomal plasmids, which represent the major pathogenicity factors of BA. However, more recent findings show that these plasmids have also been found in other closely related *Bacillus* species. In this study, we investigated the possibility of identifying species-specific and universally applicable marker peptides for BA. For this purpose, we applied a high-resolution mass spectrometry-based approach for 42 BA isolates. Along with the genomic sequencing data and by developing a bioinformatics data evaluation pipeline, which uses a database containing most of the publicly available protein sequences worldwide (UniParc), we were able to identify eleven universal marker peptides unique to BA. These markers are located on the chromosome and therefore, might overcome known problems, such as observable loss of plasmids in environmental species, plasmid loss during cultivation in the lab, and the fact that the virulence plasmids are not necessarily a unique feature of BA. The identified chromosomally encoded markers in this study could extend the small panel of already existing chromosomal targets and along with targets for the virulence plasmids, may pave the way to an even more reliable identification of BA using genomics- as well as proteomics-based techniques.

## 1. Introduction

*Bacillus anthracis* (BA) is a Gram-positive, spore-forming, rod-shaped bacterium. It is the causative agent of anthrax, an acute and often fatal disease in humans and other mammals [1,2]. Humans can become infected in various ways, e.g., when handling anthrax-infected animals, eating contaminated food, or inhaling anthrax spores. The Centers for Disease Control and Prevention (CDC) categorized BA as a potential biological agent of category A [3], and it has the potential to be used as a biological weapon. BA primarily exists in the environment as a dormant spore in the soil [4], which makes it probably the most environmentally stable category A agent overall [5].

Pathogenic strains of BA harbor two extrachromosomal plasmids—pXO1 and pXO2—which are responsible for its pathogenicity [2]. While pXO1 contains the three genes for the anthrax toxin, pXO2 contains the genes necessary to synthesize a capsule that protects the bacterium from phagocytosis [2,4,6,7,8]. The identification of BA often relies on detecting target genes located on these two virulence plasmids via nucleic acid-based assays [6,8,9].

However, BA belongs to the *Bacillus cereus* group, which is a phylogenetic cluster of closely related bacteria, including *Bacillus cereus*, *Bacillus thuringiensis*, *Bacillus mycoides*, *Bacillus pseudomycoides*, and *Bacillus weihenstephanensis* [6]. Specific identification of BA is challenging because of the high genetic similarity in this group, especially to the members *Bacillus cereus* and *Bacillus thuringiensis* [2,8]. Furthermore, recent findings show that the virulence plasmids pXO1 and pXO2 can also be present in other *Bacillus* species, leading to atypical anthrax-causing strains [10]. Even though it has been shown that the virulence plasmids are not a unique feature of BA [4,8], the target genes on these plasmids are still very important for diagnostic PCR assays [11,12,13,14].

In this study, we applied high-resolution mass spectrometry (MS) to create proteomic profiles for a collection of 42 BA isolates, mostly originating from Italy. With the thereby generated peptide profiles and the genomic sequencing data for these isolates, we first performed a hierarchical cluster analysis of the theoretical as well as the measured peptides. In the next step, we aimed to determine the presence of the virulence plasmids in the isolates directly from their genomic sequencing data. Further, we investigated the possibility of identifying universally applicable species-specific BA marker peptides from our generated genomics and proteomics data, which would enable screening for this pathogen at the proteomic level, independent of the background caused by the matrix. This was achieved by developing a bioinformatics data evaluation pipeline, which uses a database containing most of the publicly available protein sequences worldwide (UniParc) [15].

## 2. Materials and Methods

### 2.1. BA Isolates

For identification of species-specific marker peptides, we used a sample cohort of 42 BA isolates, representing genetic variation. We investigated two strains, which were isolated from cattle that died from anthrax in 2012 and 2014 in the federal state of Saxony-Anhalt in Germany, and an additional 40 strains, which were recovered from different hosts and regions in Italy. The Italian isolates represent a wide section of strains, which are different from each other in terms of location and year of isolation (from 1917–2017), as well as genetic profiles (different genotypes and lineages) (see Table S1). The German, as well as the Italian strains, have been described in previous publications [16,17,18]. Previous analyses based on classical canonical SNP typing and core genome MLST have shown that the two German strains belong to the Ames/Sterne canSNP group [19]. All strains but one belong to clade A, whereas one strain belongs to clade B (B.Br.CNEVA). The clade A strains include one strain from the Ancient A subclade (A.Br.005/006), while the remaining strains are from the TEA group (A.Br.008/011 and A.Br.011/009).

### 2.2. Bacterial Culture Preparation and Inactivation Procedure

All tested BA strains were taken from the collection of strains of IZSPB and FLI and cultivated again on 5% sheep blood agar for 24 h in aerobic conditions. Two or three colonies of every strain of BA were diluted in 2 mL tubes with 700 μL sterile deionized water and inactivated by autoclave at 121 °C for 20 min. In order to check if the inactivation was successful, 100 μL of the bacterial suspension was seeded again on 5% sheep blood agar plates after autoclavation and incubated for 24 h. If no bacterial growth was detected, the inactivation procedure was considered effective.

### 2.3. PCR

The plasmid content of the BA isolates was confirmed by real-time PCR assays. As the PCRs were performed in different labs and to different time points, the PCR assays applied in this study vary. For the procedure for isolate BA12RA1944, see Antwerpen et al. [16], while for the rest of the isolates, the protocol according to Wielinga et al. [20] was used. In brief, the presence of the BA-specific chromosomal DNA was confirmed targeting *PL3*, while for detection of pXO1 and pXO2 the genes *cya* and *capB* were used, respectively.

### 2.4. Genome Sequencing, Assembly, and Annotation of BA Isolates

As the genome sequencing was performed in different labs and to different time points, the sequencing methods applied in this study vary. Genome sequencing for the German isolates was performed as described in [16,17]. For the Italian isolates, DNA extraction and genome sequencing were performed as previously described [18]. In brief, the DNAeasy blood and tissue kit (Qiagen, Germany) was used for DNA extraction, while genome sequencing was performed on an Illumina MiSeq machine using the Nextera XT DNA Library Preparation kit (Illumina, San Diego, CA, USA) for paired-end genome library preparation. The raw sequencing reads of all data were assembled with SPAdes ([21], version 3.12.0). The assembled genomes were filtered to remove contigs with less than 5× kmer coverage and contigs shorter than 500 bp. Finally, gene annotation was performed using Prokka ([22], version 1.14), with the inclusion of a genus-specific BLAST database of BA. The genomic sequencing data has been deposited in the Sequence Read Archive public repository (https://www.ncbi.nlm.nih.gov/sra, accessed on 27 September 2022)—the corresponding IDs can be found in Table S1.

### 2.5. LC-MS/MS Analysis of BA Isolates

The proteomes of 42 autoclaved (see Section 2.1, 121 °C, 20 min) BA isolates were characterized using high-resolution MS-analysis. For this purpose, a randomized block design was applied [23], employing technical duplicates for 39 isolates as well as octuplets for 3 isolates, resulting in a total number of 102 MS runs. The samples were prepared with the iST kit (PreOmics, Martinsried, Germany), according to the manufacturer’s protocol (pelleted cells and precipitated proteins, version 2.5). For this purpose, 30 µg of each sample were pelleted for 10 min at maximum speed (21,130× *g*), and the digestion was performed using the provided trypsin-Lys-C mixture for 4 h. Desalted peptides were dried in a vacuum concentrator and dissolved in 60 µL LC-LOAD, briefly vortexed, and sonicated in a water bath for 2 min. After centrifugation for 5 min at 16,000× *g*, 6 µL were injected for nano-LC-MS/MS analysis.

LC-MS/MS analysis was performed by nano-flow reverse-phase liquid chromatography (Dionex Ultimate 3000, Thermo Scientific, Waltham, MA, USA) coupled online to a Q-Exactive HF Orbitrap mass spectrometer (Thermo Scientific), as described previously [24]. In brief, dissolved peptides were loaded onto a trapping column (µ-precolumn, 300 µm ID × 5 mm, C18 PepMap100, 5 µm, 100 Å, Thermo Scientific). The separation was performed on a PicoFrit analytical column (75 μm ID × 55 cm long, 15 µm Tip ID (New Objectives, Woburn, MA, USA)), in-house packed with 3 µm C18 resins (Reprosil-AQ Pur, Dr. Maisch, Ammerbuch-Entringen, Germany) under a controlled temperature of 50 °C. The Orbitrap was operated in a data-dependent manner: full MS (m/z range of 300 to 1750 m/z, resolution of 60,000, AGC target 1E6), followed by 12 data-dependent MS/MS scans (m/z range of 200 to 2000 m/z, resolution of 30,000, with a normalized collision energy of 25 eV, AGC target 5E5).

### 2.6. Plasmid Detection with ABRicate

For all isolates, we searched for the virulence plasmids pXO1 and pXO2 using BLAST analysis, as described in [25]. Briefly, the BLASTN based tool ABRicate (version 1.0.1, https://github.com/tseemann/abricate, accessed on 27 September 2022) was used to identify the characteristic marker genes of the two plasmids of the BA Ames ancestor with at least an 80% threshold identity and at least a 50% overlap. The marker genes are *cya*, *lef*, *pagA*, and *repX* for the pXO1 plasmid and the *capA*, *capB*, *capC*, *capD*, *capE*, and *repS* genes for the pXO2 plasmid. The plasmid was considered “detected,” if at least 50% of its marker genes were identified, “partially detected,” if less than 50% of the genes were identified, and “not detected,” if no gene was identified.

### 2.7. Data Processing and Bioinformatics Analysis

LC-MS/MS RAW files were processed using TOPP tools of the open-source library OpenMS (version 2.4) [26]. RAW files were converted to the mzML format, and, as a first preprocessing step, peak-picking and a mass calibration based on initial peptide identifications with the MS-GF+ search engine (version v2018.01.30) [27] was performed with cysteine carbamidomethylation as a fixed modification and methionine oxidation and N-terminal acetylation as variable modifications (TOPP tools: PeakPickerHiRes, MSGFPlusAdapter, InternalCalibration). Calibrated spectra were then searched again with MS-GF+, and identifications were filtered at a 1% false discovery rate. For both MS-GF+ identification runs, the union of all proteins predicted by Prokka—deduplicated using CD-HIT ([28], version 4.8.1)—was used as a database.

For hierarchical cluster analysis, peptides belonging to the 25% highest abundant peptides in at least three samples were selected. For this subset, the peptide incidence vectors were generated for each sample and used to compute a hierarchical clustering using Jaccard distance and average linkage. Peptide abundance was estimated at the MS1 level using the TOPP tool Feature Finder Identification.

### 2.8. Identification of Species-Specific Peptides

**Figure 1:**
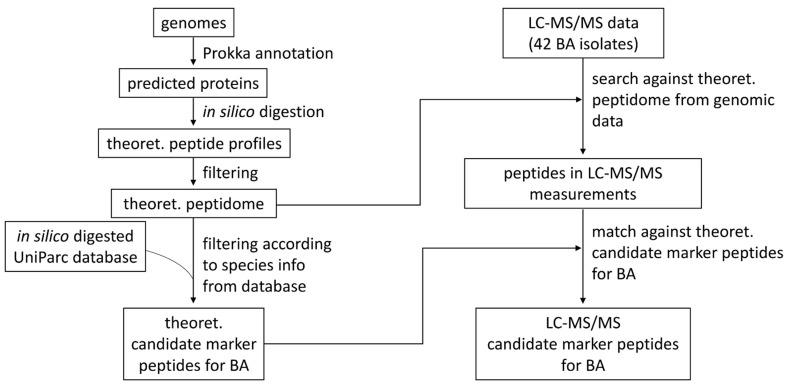
Flowchart of the bioinformatics data evaluation pipeline. The bioinformatics data evaluation comprises the processing of the genomic as well as the proteomic data. In brief, the predicted proteins for the assembled genomes were digested in silico and the resulting theoretical peptidome was matched against the active entries of an in silico digested UniParc database. The obtained theoretical candidate marker peptides were compared with the list of identified peptides from our LC-MS/MS data.

The proteins predicted for assembled genomes were digested in silico, and the resulting theoretical peptide profiles were analyzed and screened for species-specific peptides, as outlined in Figure 1.

In silico digestion was performed with trypsin and without missed cleavages, retaining all peptides of 8–30 amino acids length. Next, we determined the core peptidome, i.e., the set of peptides occurring in the protein predictions of every sample. From this set of 52,707 peptides, a subset of 789 species-specific peptides was extracted. For this purpose, peptides were matched against all in silico digested active entries in the UniParc database (18/01/2022, ~458 million sequences). To enhance specificity, a conservative approach was chosen, and the isomeric amino acids, leucine and isoleucine, and isobaric amino acids, glutamine and lysine, were treated as equivalent during the search. Peptides not matching any other species than BA were considered species-specific. LC-MS/MS peptide identifications were finally matched against the set of species-specific peptides and filtered according to their sample coverage (fraction of samples containing the peptide identification) and their median percentile rank (percentage of peptide identifications with lower intensity) over all covering samples. Species-specific identifications were filtered at a sample coverage of 50% and a median percentile rank of 50 in order to ensure a good detectability from the LC-MS samples.

The processed MS raw and KNIME output files have been deposited to the ProteomeXchange Consortium and can be found in the PRIDE repository (http://proteomecentral.proteomexchange.org, accessed on 27 September 2022) PXD036243. The KNIME postprocessing workflows and the implementations for candidate peptide filtering are available upon request.

## 3. Results

High-resolution MS was used to create proteomic profiles for a collection of BA isolates. Using the thereby generated proteomic data, as well as our genomic sequencing data for these isolates, we first performed hierarchical cluster analyses of the theoretical and the measured peptide profiles. Further, we tested the possibility of determining the presence of the virulence plasmids in the isolates directly from their genomic sequencing data. As a final step, we investigated the possibility of identifying species-specific BA marker peptides from our genomic as well as proteomic data. These markers should additionally fulfill the requirement that they are universally applicable, and would therefore enable screening for this pathogen on the proteomic level, independent of the background. To ensure this, we developed a bioinformatics data evaluation pipeline, which uses a database comprising most of the publicly available protein sequences worldwide (UniParc).

### 3.1. Cluster Analyses

**Figure 2:**
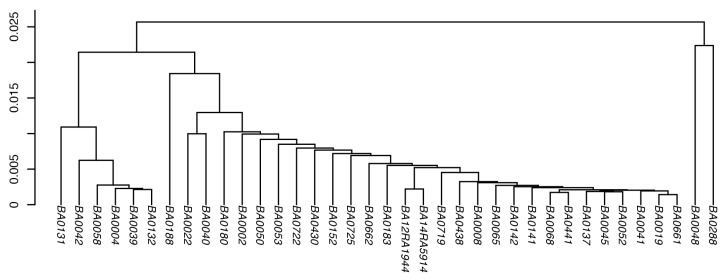
Cluster analysis based on the theoretical peptide profiles of the BA isolates. A cluster analysis was performed using the predicted peptides, created in silico from the genomic data of the BA isolates. For hierarchical clustering, Jaccard distance (*y*-axis) and average linkage were used.

**Figure 3:**
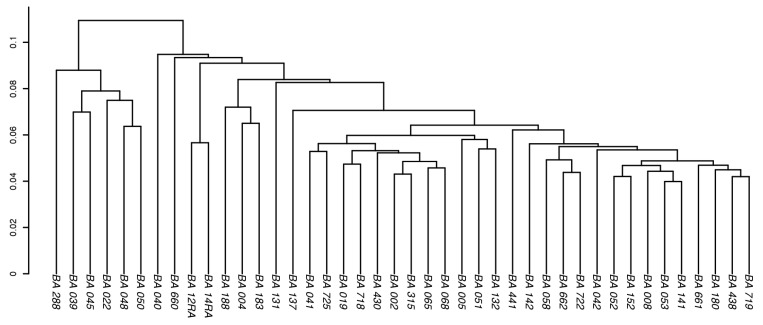
Cluster analysis of the measured peptide profiles of the BA isolates. A cluster analysis was also performed for the measured proteomic data of 42 BA isolates. On the *y*-axis, the cluster distance is given according to Jaccard.

Cluster analyses were carried out for both the theoretical and the measured peptide profiles (see Figure 2 and Figure 3) to investigate any fine structure in our data that would possibly allow distinguishing subgroups of the isolates from each other. Both analyses show that the BA isolates cluster closely together and are very similar to each other; thus, no subgroups could be defined.

### 3.2. Analysis of Plasmid Content

In the next step, we aimed to determine the presence of the virulence plasmids directly from the genomic sequencing data of 37 BA isolates (see Table S2). For this purpose, we searched in our sequencing data for four marker genes corresponding to pXO1 and for six genes corresponding to pXO2. As a result of this analysis, in five isolates none, and in two isolates not all of the marker genes for pXO1 were detected. Further, in one isolate, no markers, neither for pXO1 nor the pXO2 plasmid, were detected, while in the rest of the isolates, both of the virulence plasmids were detected. Comparing these results with the outcome of the PCR analysis showed that isolates which did not contain a plasmid, according to the PCR results, were also negative in our bioinformatics analysis. On the other hand, none (see isolate BA0132) or not all (see isolates BA0002 and BA0183) of the marker genes for the pXO1 plasmid were detected in the sequencing data of three isolates, even though the corresponding PCR data revealed positive results for this plasmid.

### 3.3. Identification of Species-Specific Candidate Marker Peptides for BA

Due to the high similarity between the BA isolates on the genomic, as well as on the proteomic level, potential candidate marker peptides were sought at the species level for BA. For this purpose, a bioinformatic approach was applied that only considered the identified peptides from the LC-MS/MS data, which were found only in the species BA and in no other species present in the UniParc database (see Figure 1). In this way, we were able to identify eleven species-specific candidate marker peptides for BA (see Table 1). Exemplary MS2 spectra for the peptides are provided in the Supplementary Materials section of this manuscript. Seven of these marker candidates are present in at least 80% of all LC-MS/MS measurements, and only two peptides occur in every measurement. These marker peptides were also identified in the Prokka annotations of all sequenced isolates. Among the proteins associated with these peptides are proteins belonging to the spores and metabolic enzymes, as well as a ribosomal protein of BA.

## 4. Discussion

In this study, we used a combined approach of high-resolution MS and genomic sequencing for a collection of BA isolates to identify species-specific and universally applicable marker peptides for BA. Based on the proteomic and genomic datasets of 42 individual BA isolates, cluster analyses were created using the theoretical, as well as the LC-MS/MS, peptide profiles (see Figure 2 and Figure 3), respectively. All isolates clustered very closely together and thus, are very similar to each other at the proteomic and genomic levels. This is most likely because BA is known to be one of the most molecularly homogenous bacteria, displaying little genetic variation [4,7,8,19,29,30,31]. Additionally, most of the BA isolates used in this study originate from Italy (see Table S1), where majority of occurring BA strains are genetically so similar that they are believed to have descended and evolved from a local common ancestral strain [30].

As a next step, we investigated the possibility of determining the presence of the virulence plasmids in the isolates directly from their genomic sequencing data. Most of the strains in this study were isolated from lethal anthrax outbreaks in animals (see Table S1), meaning that they were fully virulent at the time of their isolation and therefore, possessed both virulence plasmids. However, the analysis of our genomic sequencing data revealed that both virulence plasmids could not be detected in all isolates (see Table S2). These results were, in most cases, in agreement with the PCR data of the strains. The absence of the plasmids in some of the isolates might result from a loss of one or both plasmids during various cultivation processes in the lab. In this context, it is interesting that the oldest investigated strain in this study, which was isolated in 1917, was also the only isolate that did not contain any virulence plasmids (see Tables S1 and S2). However, there was also one isolate that showed different results between both analyses. While the PCR result for the strain BA0132 detected the presence of both virulence plasmids, the analysis of the sequencing data did not detect any marker genes for pXO1. This difference between both analyses is most likely a consequence of the fact that the genomic sequencing of the isolates was performed later than the PCR analysis. It is reasonable to assume that this isolate lost its pXO1 plasmid in the meantime. Indeed, reanalyzing this isolate via PCR confirmed this assumption, thus no pXO1 plasmid could be detected for this strain. Furthermore, not all the marker genes for pXO1 were detected in the sequencing data of the two isolates BA0002 and BA0183 (see Table S2). Since for the isolate BA0183, three out of four marker genes were detected for pXO1, it is safe to assume that the complete plasmid was present in this isolate. For the isolate BA0002, only one out of four genes for pXO1 was detected. For closer inspection, we aligned the assembled genome of this isolate against the reference sequence of pXO1. Except for the marker gene *repX*, there were also other parts of the plasmid present, but they did not contain the three marker genes *cya*, *lef*, and *pagA*. Therefore, the classification “partially detected” for the presence of the plasmid pXO1 in this isolate is most likely due to the untargeted nature of the applied genomic sequencing method, as well as the subsequent genome assembly, than to the presence of the plasmid itself. Keeping in mind the limitations of this method, detecting the virulence plasmids of BA directly from its sequencing data proved to be a viable tool. Interestingly, when comparing these results with the cluster analysis of the theoretical peptide profiles, it could be seen that the Italian isolates missing the pXO2, or both virulence plasmids (see Figure 2, isolates BA0004, BA0039, BA0042, BA0058, BA0132, and BA0131, respectively), form a cluster, which separates them from the Italian isolates possessing both virulence plasmids. This comparison is also in agreement with the absence of pXO1 in the isolate BA0132, while the isolate BA0002 possesses both plasmids, as both isolates cluster according to their plasmid content.

Even though BA shows little genetic variation [4,8,19,29,30], the identification of such marker peptides proved to be challenging. BA is a member of the *Bacillus cereus* group [4,6,7,8,29,32,33], a group of closely related Gram-positive, endospore-forming bacteria [29,33]. The specific identification of BA is hampered due to the high genetic similarity in this group [2,6,8,29,32,33]—especially to the two members *Bacillus cereus* and *Bacillus thuringiensis*, which have a sequence similarity of over 99% compared to BA [8]. The fact that these two species occur ubiquitously further complicates the identification of BA [2,8,33,34]. The main difference between these three species is the presence of the two extrachromosomal virulence plasmids pXO1 and pXO2 in BA, which are responsible for its pathogenicity [2,4,6,7,8]. However, more recent findings have shown that these two virulence plasmids are not a unique feature of BA [4,8]. There has been an increasing number of reports of so-called atypical *Bacillus cereus* strains that caused anthrax-like diseases in humans and other mammals [12,13,29,35,36,37]. These strains are defined by their *Bacillus cereus* chromosomal DNA and the presence of virulence plasmids that show a very high level of similarity with the anthrax virulence plasmids pXO1 and pXO2 [29]. Thus, the anthrax toxin produced by the atypical *Bacillus cereus* strains is not significantly different from that produced by BA, and infection with these strains may lead to both similar symptoms and mortality rates compared to anthrax caused by BA [29]. Despite these high similarities, we were able to identify eleven species-specific candidate marker peptides for BA, which allow for differentiating BA from all other species, including the closely related *Bacillus cereus* groups (see Table 1). These eleven markers are all chromosomally encoded and tie in with recent studies reporting the interest of researchers in chromosome-encoded genes, which would be preferable for the specific detection of BA, due to occasionally observed losses of virulence plasmids within environmental species and the occurrence of virulence plasmids in atypical *Bacillus cereus* strains [6,8,38,39].

Research in the literature revealed that three of eleven identified candidate marker peptides from our study overlap with previously described proteomic markers [2,8]. While two of those markers are an exact match, the third shows only a partial overlap of the peptide sequence, since this peptide was created by enzymatic digestion using a protease other than trypsin (Glu-C) (see Table 1, peptides with annotations short-chain-enoyl-CoA hydratase, 50S ribosomal protein L5, and small, acid-soluble spore protein gamma-type, respectively). However, in this case, the associated protein, as well as the peptide position within this protein, both match. Thus, the peptide belonging to the Prokka annotation short-chain enoyl-CoA hydratase (see Table 1) has already been reported by Misra et al. [2], who developed a discovery pipeline for elucidating peptide biomarkers exemplarily for the pathogen BA. Two other potential marker peptides overlap with identified BA spore markers from a study by Chenau et al. [8], who undertook comparative proteomics analyses of BA, *Bacillus cereus*, and *Bacillus thuringiensis* spores to identify proteoforms unique to BA. The peptide markers in question are the peptides associated with the annotations 50S ribosomal protein L5 (RL5) and small, acid-soluble spore protein gamma-type (SASP-gamma). The protein RL5 is a ribosomal protein, which comprises up to one-fifth of the total protein content in bacterial cells [8,40]. In BA, ribosomal proteins, such as RL5, can be expressed in both vegetative cells, as well as germinating spores, so this marker is not necessarily a spore marker [8]. This marker was additionally mentioned by Rajoria et al. [41] as a suitable BA marker for the detection of biological warfare agents.

The protein SASP-gamma belongs to a group of small proteins that are only formed during sporulation [8]. These small, acid-soluble spore proteins (SASPs) are the predominant class of proteins in the spore core of BA and they serve, among other functions, to protect the spore chromosome from chemical, enzymatic, and UV damage (type alpha and beta), and likely play a role in osmoregulation (type gamma) [42]. Their concentration within the spores is so high that even the presence of a small number of spores within a large number of vegetative cells would result in a strong SASPs signal [8].

The fact that we were able to identify these two marker peptides, belonging to the annotations RL5 and SASP-gamma, is also directly related to the results of the study of Chenau et al. [8], meaning that both their identified spore markers are more universally applicable than just to distinguish BA from *Bacillus cereus* and *Bacillus thuringiensis* spores.

Upon further inspection of the marker candidates, there was another spore-associated peptide identified belonging to the protein spore germination protein YaaH (see Table 1). This protein is present in the spores of BA and is required for their germination in vegetative cells [43,44]. The fact that these three potential marker candidates, belonging to the proteins RL5, SASP-gamma, and spore germination protein YaaH, are spore-associated may explain why they could not be found in all LC-MS/MS measurements (see Table 1). It is conceivable that not all BA isolates contained spores, or only to such a small extent that they were not identified during proteome profiling.

Considering all identified markers in this study, it was striking that only two peptides were detected in all the measured samples (see Table 1). However, it should also be kept in mind that these peptides are potential candidate marker peptides that could be used in order to create a targeted MS method. In an ideal case, screening of all isolates with such a method could result in the detection of some of these eleven markers in all samples. Furthermore, a large-scale study would be needed to additionally verify the presence of the herein identified marker peptides in BA strains from different geographical locations.

As discussed previously, while methods targeting the virulence plasmids only verify the presence of the plasmids, species-specific molecular identification of BA can be achieved by using chromosomal targets [39]. In this context, it is especially interesting that the genes coding for the here identified potential marker candidates are chromosomally localized. This may offer the possibility to develop genomic assays, which could complement the small panel of already existing chromosomal markers, such as *dhp61* [45], *PL3* [20], or a novel approach using the multi-copy 16S rRNA gene [39], which are used in addition to markers for the virulence plasmids. Indeed, the research revealed that the *sspE* gene—coding for the protein SASP-gamma—was already used, in addition to two genes for the virulence plasmids, to specifically identify BA using PCR assays [46,47].

In summary, this study demonstrates that it is possible to identify potential candidate marker peptides capable of distinguishing BA from all other species sequenced to date, including all members of the closely related *Bacillus cereus* group. These marker peptides were identified using a holistic bioinformatics workflow that considered a broad database (UniParc) as a background; therefore, these markers are universally applicable. The data evaluation workflow created in this study could also be adapted to search against a specific background of interest, e.g., a sample matrix including its most common microorganisms and contaminants, to identify corresponding BA markers. Another exemplary application would be to identify anthrax-causing marker peptides, considering that the anthrax risk is no longer only restricted to BA. Nevertheless, the here identified potential marker candidates could be used to establish a targeted MS method to specifically identify BA on a proteomic level. For this purpose, this marker panel could be complemented by BA spore markers, e.g., from Chenau et al. [8]. Moreover, our identified chromosomally encoded markers could complement the small number of already existing chromosomal targets and along with targets for the virulence plasmids may pave the way to an even more reliable identification of BA using genomic assays.

## Figures and Tables

**Table 1:** Identified species-specific candidate marker peptides for BA. The following eleven candidate marker peptides were found, which are all unique to the species BA and cannot be found in any other species occurring in the UniParc database. In addition to the peptide sequences, the corresponding Prokka annotations are given, as well as the median intensity percentile rank. The last column shows the percentage of measurements in which these marker peptides were identified. The marker peptides listed here are filtered as follows: they appear in all Prokka annotations, have a median intensity percentile rank of at least 50, and appear in at least 50% of the LC-MS/MS measurements.

Peptide Sequence	Prokka Annotation	Median Intensity Percentile Rank	Identified in LC-MS/MS Measurements (%)
QLSDVAEEDVNR	Short-chain-enoyl-CoA hydratase	91.20	100.00
EINGQAQTQTTVTETK	putative peptidoglycan endopeptidase LytE	52.24	100.00
FQMGNQHYSIDVHK	hypothetical protein	85.65	97.06
FFETHDYK	Spore germination protein YaaH	84.96	86.27
TPGHGIYTGIK	Asparagine synthetase glutamine-hydrolyzing 3	73.83	83.33
NIAQGAALATDTK	p-aminobenzoyl-glutamate hydrolase subunit B	59.47	81.37
TLDNAVEELTQITGQK	50S ribosomal protein L5	62.58	81.37
EDSPLVTLTGK	PhoH-like protein	63.61	75.49
NSSHWAEEDTILSGVK	N-acetyltransferase YodP	64.46	73.53
AQASGASIQSTNASYGTEFATETDVHAVK	Small, acid-soluble spore protein gamma-type	92.41	66.67
VLTSGQEAVSHNFLGDK	putative 3-hydroxyacyl-CoA dehydrogenase	61.27	55.88

## Data Availability

The genomic datasets used in this study can be found in the Sequence Read Archive public repository (https://www.ncbi.nlm.nih.gov/sra, accessed on 27 September 2022)—the repository IDs can be found in Table S1. The proteomics data can be found via the PRIDE partner repository (http://proteomecentral.proteomexchange.org, accessed on 27 September 2022) under the corresponding ID PXD036243.

## Supplementary Materials

The following supporting information can be downloaded at: https://www.mdpi.com/article/10.3390/life12101549/s1, Table S1: Overview of all analyzed BA isolates in this study; Table S2: Overview of BA isolates and their virulence plasmid content, Supplementary Mass Spectra.pdf.

## References

[1] Dixon T.C., Meselson M., Guillemin J., Hanna P.C. (1999). Anthrax. N. Engl. J. Med..

[2] Misra R.V., Ahmod N.Z., Parker R., Fang M., Shah H., Gharbia S. (2012). Developing an Integrated Proteo-Genomic Approach for the Characterisation of Biomarkers for the Identification of *Bacillus anthracis*. J. Microbiol. Methods.

[3] Rotz L.D., Khan A.S., Lillibridge S.R., Ostroff S.M., Hughes J.M. (2002). Public Health Assessment of Potential Biological Terrorism Agents. Emerg. Infect. Dis..

[4] Kolstø A.-B., Tourasse N.J., Økstad O.A. (2009). What Sets *Bacillus anthracis* Apart from Other *Bacillus* Species?. Annu. Rev. Microbiol..

[5] Chenau J., Fenaille F., Ezan E., Morel N., Lamourette P., Goossens P.L., Becher F. (2011). Sensitive Detection of *Bacillus anthracis* Spores by Immunocapture and Liquid Chromatography–Tandem Mass Spectrometry. Anal. Chem..

[6] Zasada A.A. (2020). Detection and Identification of *Bacillus anthracis*: From Conventional to Molecular Microbiology Methods. Microorganisms.

[7] Pena-Gonzalez A., Rodriguez-R L.M., Marston C.K., Gee J.E., Gulvik C.A., Kolton C.B., Saile E., Frace M., Hoffmaster A.R., Konstantinidis K.T. (2018). Genomic Characterization and Copy Number Variation of *Bacillus anthracis* Plasmids PXO1 and PXO2 in a Historical Collection of 412 Strains. mSystems.

[8] Chenau J., Fenaille F., Caro V., Haustant M., Diancourt L., Klee S.R., Junot C., Ezan E., Goossens P.L., Becher F. (2014). Identification and Validation of Specific Markers of *Bacillus anthracis* Spores by Proteomics and Genomics Approaches*. Mol. Cell. Proteom..

[9] Irenge L.M., Gala J.-L. (2012). Rapid Detection Methods for *Bacillus anthracis* in Environmental Samples: A Review. Appl. Microbiol. Biotechnol..

[10] Irenge L.M., Bearzatto B., Ambroise J., Gala J.-L., Rasko D. (2020). Complete Genome Sequence of an Environmental *Bacillus cereus* Isolate Belonging to the *Bacillus anthracis* Clade. Microbiol. Resour. Announc..

[11] Pannucci J., Okinaka R.T., Sabin R., Kuske C.R. (2002). *Bacillus anthracis* PXO1 Plasmid Sequence Conservation among Closely Related Bacterial Species. J. Bacteriol..

[12] Hoffmaster A.R., Ravel J., Rasko D.A., Chapman G.D., Chute M.D., Marston C.K., De B.K., Sacchi C.T., Fitzgerald C., Mayer L.W. (2004). Identification of Anthrax Toxin Genes in a *Bacillus cereus* Associated with an Illness Resembling Inhalation Anthrax. Proc. Natl. Acad. Sci. USA.

[13] Hoffmaster A.R., Hill K.K., Gee J.E., Marston C.K., De B.K., Popovic T., Sue D., Wilkins P.P., Avashia S.B., Drumgoole R. (2006). Characterization of *Bacillus cereus* Isolates Associated with Fatal Pneumonias: Strains Are Closely Related to *Bacillus anthracis* and Harbor *B. anthracis* Virulence Genes. J. Clin. Microbiol..

[14] Klee S.R., Nattermann H., Becker S., Urban-Schriefer M., Franz T., Jacob D., Appel B. (2006). Evaluation of Different Methods to Discriminate *Bacillus anthracis* from Other Bacteria of the *Bacillus cereus* Group. J. Appl. Microbiol..

[15] Leinonen R., Diez F.G., Binns D., Fleischmann W., Lopez R., Apweiler R. (2004). UniProt Archive. Bioinformatics.

[16] Antwerpen M., Elschner M., Gaede W., Schliephake A., Grass G., Tomaso H. (2016). Genome Sequence of *Bacillus anthracis* Strain Stendal, Isolated from an Anthrax Outbreak in Cattle in Germany. Genome Announc..

[17] Elschner M.C., Busch A., Schliephake A., Gaede W., Zuchantke E., Tomaso H. (2017). High-Quality Genome Sequence of *Bacillus anthracis* Strain 14RA5914 Isolated during an Outbreak in Germany in 2014. Genome Announc..

[18] Abdel-Glil M.Y., Chiaverini A., Garofolo G., Fasanella A., Parisi A., Harmsen D., Jolley K.A., Elschner M.C., Tomaso H., Linde J. (2021). A Whole-Genome-Based Gene-by-Gene Typing System for Standardized High-Resolution Strain Typing of *Bacillus anthracis*. J. Clin. Microbiol..

[19] Van Ert M.N., Easterday W.R., Huynh L.Y., Okinaka R.T., Hugh-Jones M.E., Ravel J., Zanecki S.R., Pearson T., Simonson T.S., U’Ren J.M. (2007). Global Genetic Population Structure of *Bacillus anthracis*. PLoS ONE.

[20] Wielinga P.R., Hamidjaja R.A., Ågren J., Knutsson R., Segerman B., Fricker M., Ehling-Schulz M., de Groot A., Burton J., Brooks T. (2011). A Multiplex Real-Time PCR for Identifying and Differentiating *B. anthracis* Virulent Types. Int. J. Food Microbiol..

[21] Bankevich A., Nurk S., Antipov D., Gurevich A.A., Dvorkin M., Kulikov A.S., Lesin V.M., Nikolenko S.I., Pham S., Prjibelski A.D. (2012). SPAdes: A New Genome Assembly Algorithm and Its Applications to Single-Cell Sequencing. J. Comput. Biol..

[22] Seemann T. (2014). Prokka: Rapid Prokaryotic Genome Annotation. Bioinformatics.

[23] Oberg A.L., Vitek O. (2009). Statistical Design of Quantitative Mass Spectrometry-Based Proteomic Experiments. J. Proteome Res..

[24] Witt N., Andreotti S., Busch A., Neubert K., Reinert K., Tomaso H., Meierhofer D. (2020). Rapid and Culture Free Identification of Francisella in Hare Carcasses by High-Resolution Tandem Mass Spectrometry Proteotyping. Front. Microbiol..

[25] Liu Y., Lai Q., Göker M., Meier-Kolthoff J.P., Wang M., Sun Y., Wang L., Shao Z. (2015). Genomic Insights into the Taxonomic Status of the *Bacillus cereus* Group. Sci. Rep..

[26] Röst H.L., Sachsenberg T., Aiche S., Bielow C., Weisser H., Aicheler F., Andreotti S., Ehrlich H.-C., Gutenbrunner P., Kenar E. (2016). OpenMS: A Flexible Open-Source Software Platform for Mass Spectrometry Data Analysis. Nat. Methods.

[27] Kim S., Pevzner P.A. (2014). MS-GF+ Makes Progress towards a Universal Database Search Tool for Proteomics. Nat. Commun..

[28] Fu L., Niu B., Zhu Z., Wu S., Li W. (2012). CD-HIT: Accelerated for Clustering the next-Generation Sequencing Data. Bioinformatics.

[29] Baldwin V.M. (2020). You Can’t *B. cereus*—A Review of *Bacillus cereus* Strains That Cause Anthrax-like Disease. Front. Microbiol..

[30] Rondinone V., Serrecchia L., Parisi A., Fasanella A., Manzulli V., Cipolletta D., Galante D. (2020). Genetic Characterization of *Bacillus anthracis* Strains Circulating in Italy from 1972 to 2018. PLoS ONE.

[31] Edwards K.A., Clancy H.A., Baeumner A.J. (2006). *Bacillus anthracis*: Toxicology, Epidemiology and Current Rapid-Detection Methods. Anal. Bioanal. Chem..

[32] (2020). *Bacillus cereus*-Bakterien in Lebensmitteln können Magen-Darm-Erkrankungen verursachen: Stellungnahme Nr. 048/2020 des BfR vom 30. Oktober 2020. BfR-Stellungnahmen.

[33] Pfrunder S., Grossmann J., Hunziker P., Brunisholz R., Gekenidis M.-T., Drissner D. (2016). *Bacillus cereus* Group-Type Strain-Specific Diagnostic Peptides. J. Proteome Res..

[34] Santos E.N., Menezes L.P., Dolabella S.S., Santini A., Severino P., Capasso R., Zielinska A., Souto E.B., Jain S. (2022). *Bacillus thuringiensis*: From Biopesticides to Anticancer Agents. Biochimie.

[35] Hoffmann C., Zimmermann F., Biek R., Kuehl H., Nowak K., Mundry R., Agbor A., Angedakin S., Arandjelovic M., Blankenburg A. (2017). Persistent Anthrax as a Major Driver of Wildlife Mortality in a Tropical Rainforest. Nature.

[36] Klee S.R., Özel M., Appel B., Boesch C., Ellerbrok H., Jacob D., Holland G., Leendertz F.H., Pauli G., Grunow R. (2006). Characterization of *Bacillus anthracis*-Like Bacteria Isolated from Wild Great Apes from Côte d’Ivoire and Cameroon. J. Bacteriol..

[37] Marston C.K., Ibrahim H., Lee P., Churchwell G., Gumke M., Stanek D., Gee J.E., Boyer A.E., Gallegos-Candela M., Barr J.R. (2016). Anthrax Toxin-Expressing *Bacillus cereus* Isolated from an Anthrax-like Eschar. PLoS ONE.

[38] Ågren J., Hamidjaja R.A., Hansen T., Ruuls R., Thierry S., Vigre H., Janse I., Sundström A., Segerman B., Koene M. (2013). In Silico and in Vitro Evaluation of PCR-Based Assays for the Detection of *Bacillus anthracis* Chromosomal Signature Sequences. Virulence.

[39] Braun P., Nguyen M.D.-T., Walter M.C., Grass G. (2021). Ultrasensitive Detection of *Bacillus anthracis* by Real-Time PCR Targeting a Polymorphism in Multi-Copy 16S RRNA Genes and Their Transcripts. Int. J. Mol. Sci..

[40] Bremer H., Dennis P.P. (2008). Modulation of Chemical Composition and Other Parameters of the Cell at Different Exponential Growth Rates. EcoSal Plus.

[41] Rajoria S., Sabna S., Babele P., Kumar R.B., Kamboj D.V., Kumar S., Alam S.I. (2020). Elucidation of Protein Biomarkers for Verification of Selected Biological Warfare Agents Using Tandem Mass Spectrometry. Sci. Rep..

[42] Jagtap P., Michailidis G., Zielke R., Walker A.K., Patel N., Strahler J.R., Driks A., Andrews P.C., Maddock J.R. (2006). Early Events of *Bacillus anthracis* Germination Identified by Time-Course Quantitative Proteomics. Proteomics.

[43] Lambert E.A., Popham D.L. (2008). The *Bacillus anthracis* SleL (YaaH) Protein Is an N-Acetylglucosaminidase Involved in Spore Cortex Depolymerization. J. Bacteriol..

[44] Lambert E.A., Sherry N., Popham D.L. (2012). In Vitro and in Vivo Analyses of the *Bacillus anthracis* Spore Cortex Lytic Protein SleL. Microbiology.

[45] Antwerpen M.H., Zimmermann P., Bewley K., Frangoulidis D., Meyer H. (2008). Real-Time PCR System Targeting a Chromosomal Marker Specific for *Bacillus anthracis*. Mol. Cell. Probes.

[46] Kim K., Seo J., Wheeler K., Park C., Kim D., Park S., Kim W., Chung S.-I., Leighton T. (2005). Rapid Genotypic Detection of *Bacillus anthracis* and the *Bacillus cereus* Group by Multiplex Real-Time PCR Melting Curve Analysis. FEMS Immunol. Med. Microbiol..

[47] Janse I., Hamidjaja R.A., Bok J.M., van Rotterdam B.J. (2010). Reliable Detection of *Bacillus anthracis*, Francisella Tularensis and Yersinia Pestis by Using Multiplex QPCR Including Internal Controls for Nucleic Acid Extraction and Amplification. BMC Microbiol..

