# Supplementary material for: Identification of Universally Applicable and Species-Specific Marker Peptides for Bacillus anthracis
Source: Life (Basel). 2022 Oct 6;12(10):1549. doi: 10.3390/life12101549 (PMC9605612; doi:10.3390/life12101549)
Supplement: Supplementary file 1 [file life-12-01549-s001.zip › Supplementary Mass Spectra.pdf]

Supplementary Mass Spectra: On the following pages, a mass spectrum is given for each of the eleven species-specific candidate marker peptides for BA. For every annotated spectrum the identified fragments, peptide sequence, as well as the respective Prokka annotation are given, together with information regarding the precursor m/z, its charge and search engine score.

QLSDVAEEDVNR, Short-chain-enoyl-CoA hydratase

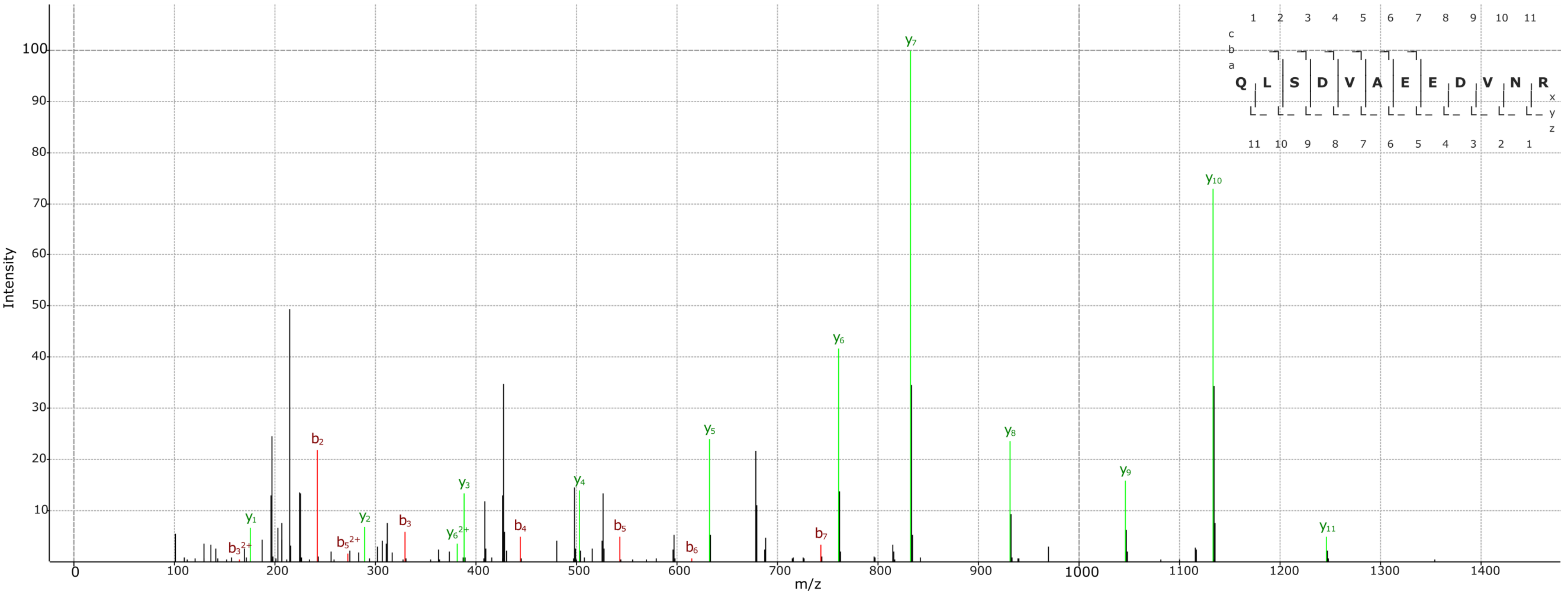

Precursor m/z:	687.8
Peptide charge:	2
Score:	9.53E-16

EINGQAQTQTTVTETK, putative peptidoglycan endopeptidase LytE

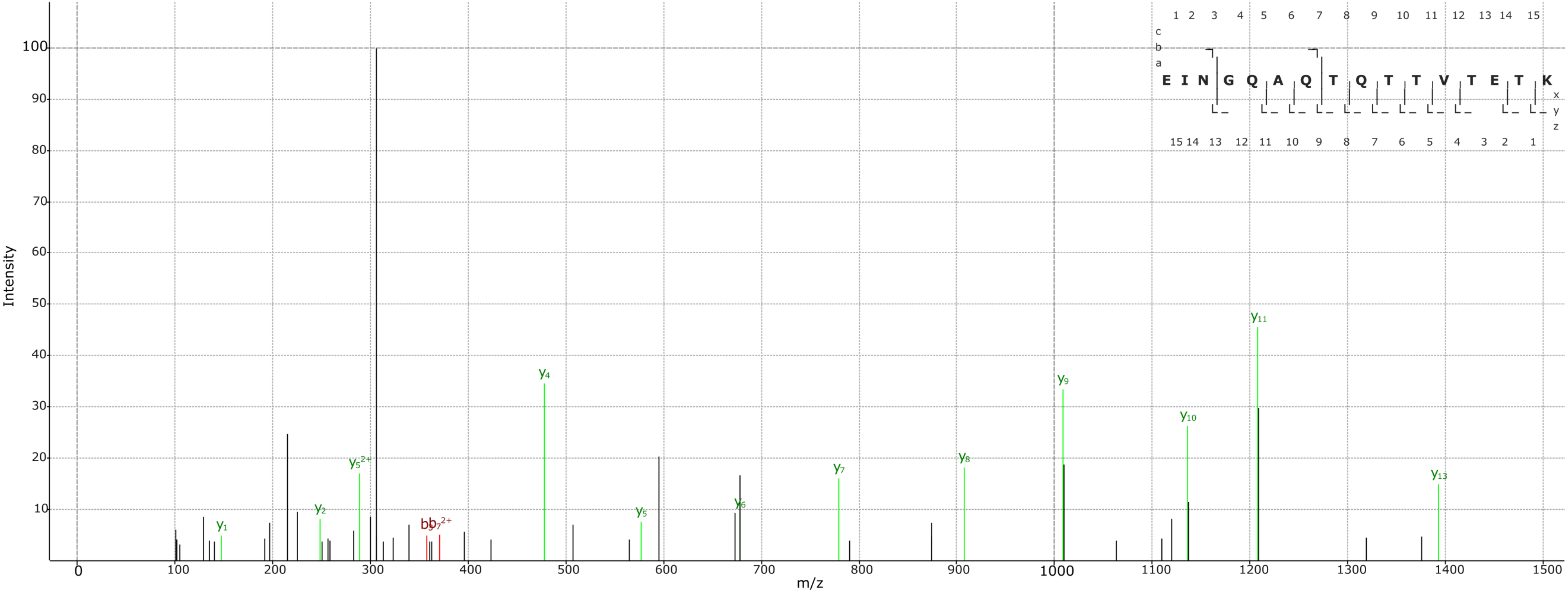

Precursor m/z:	874.9
Peptide charge:	2
Score:	8.49E-19

FQMGNQHYSIDVHK, hypothetical protein

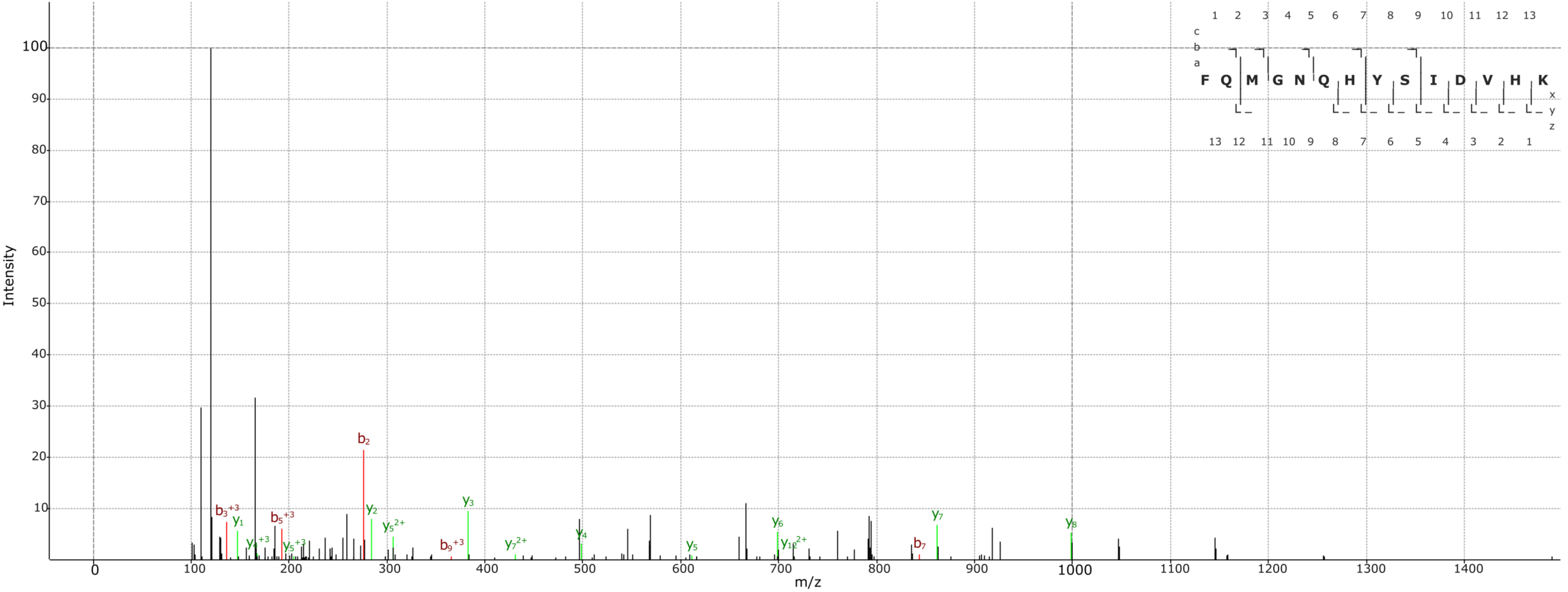

Precursor m/z:	568.6
Peptide charge:	3
Score:	6.53E-15

FFETHDYK, Spore germination protein YaaH

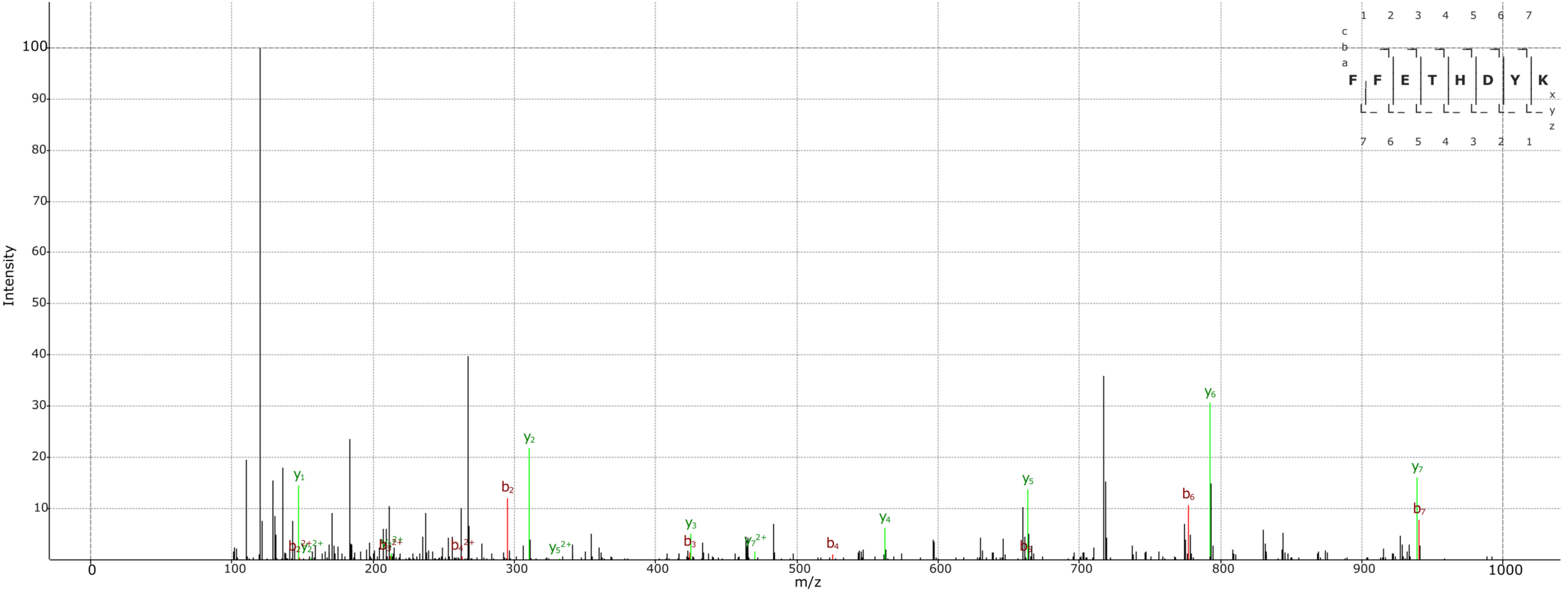

Precursor m/z:	544.3
Peptide charge:	2
Score:	4.30E-12

TPGHGIYTGIK, Asparagine synthetase [glutamine-hydrolyzing] 3

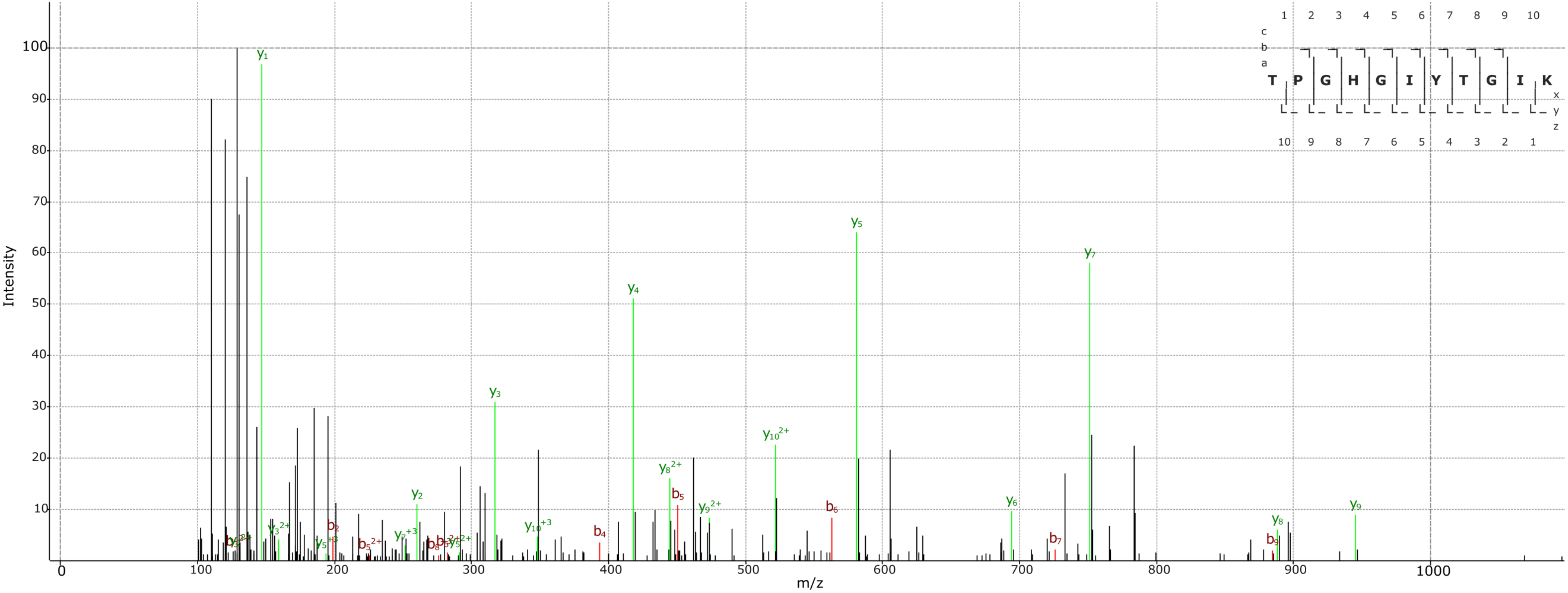

Precursor m/z:	381.9
Peptide charge:	3
Score:	7.04E-15

NIAQGAALATDTK, p-aminobenzoyl-glutamate hydrolase subunit B

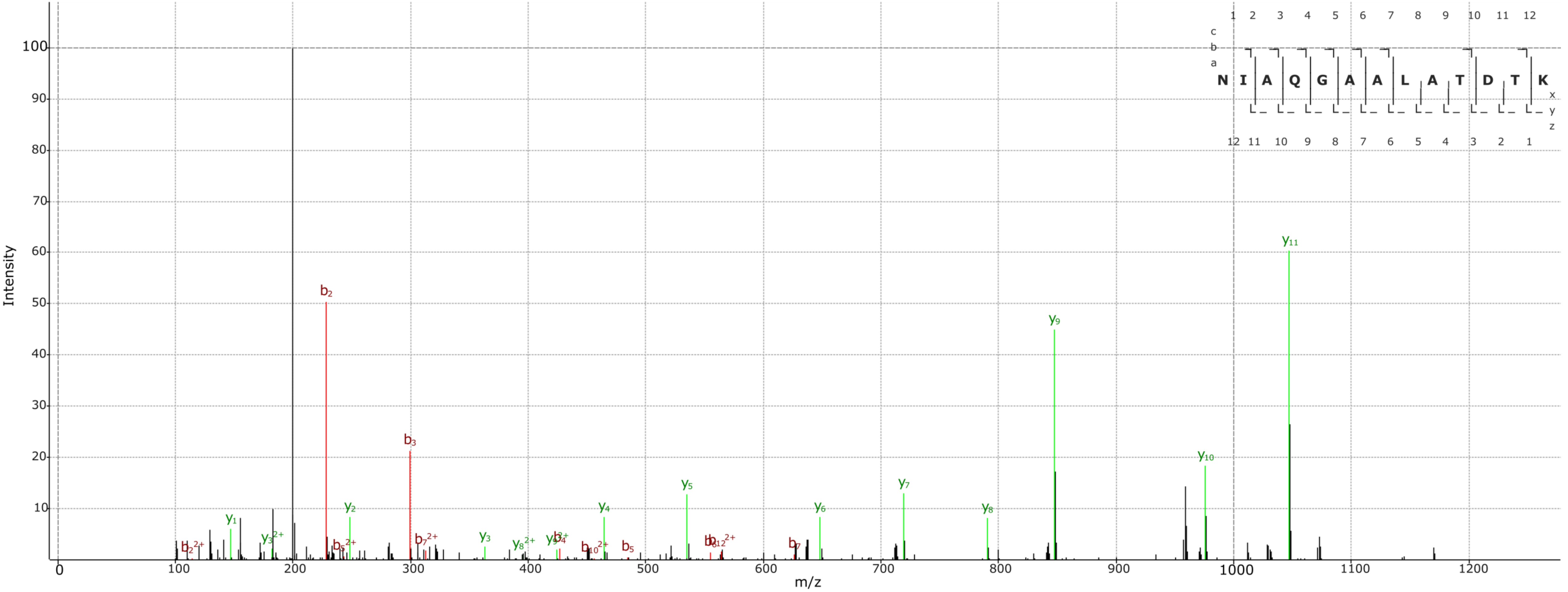

Precursor m/z:	637.3
Peptide charge:	2
Score:	1.35E-15

TLDNAVEELTQITGQK, 50S ribosomal protein L5

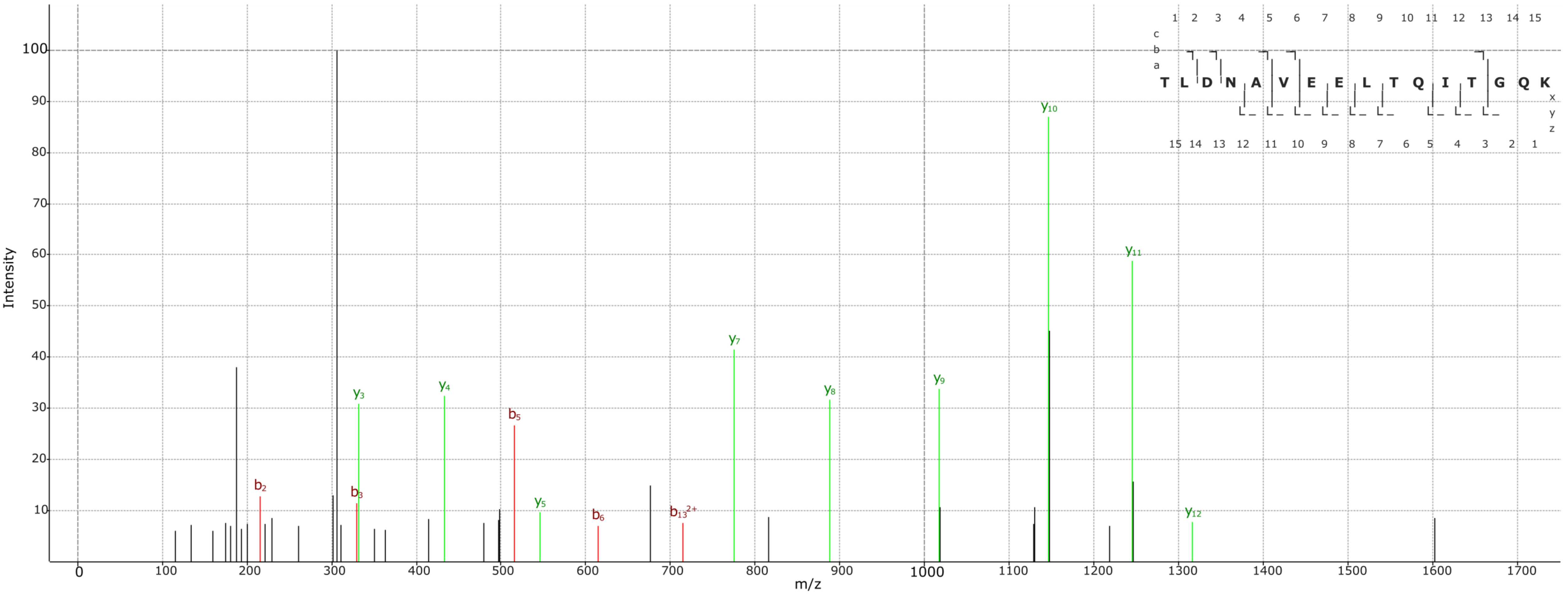

Precursor m/z:	880.5
Peptide charge:	2
Score:	3.60E-15

EDSPLVTLTGK, PhoH-like protein

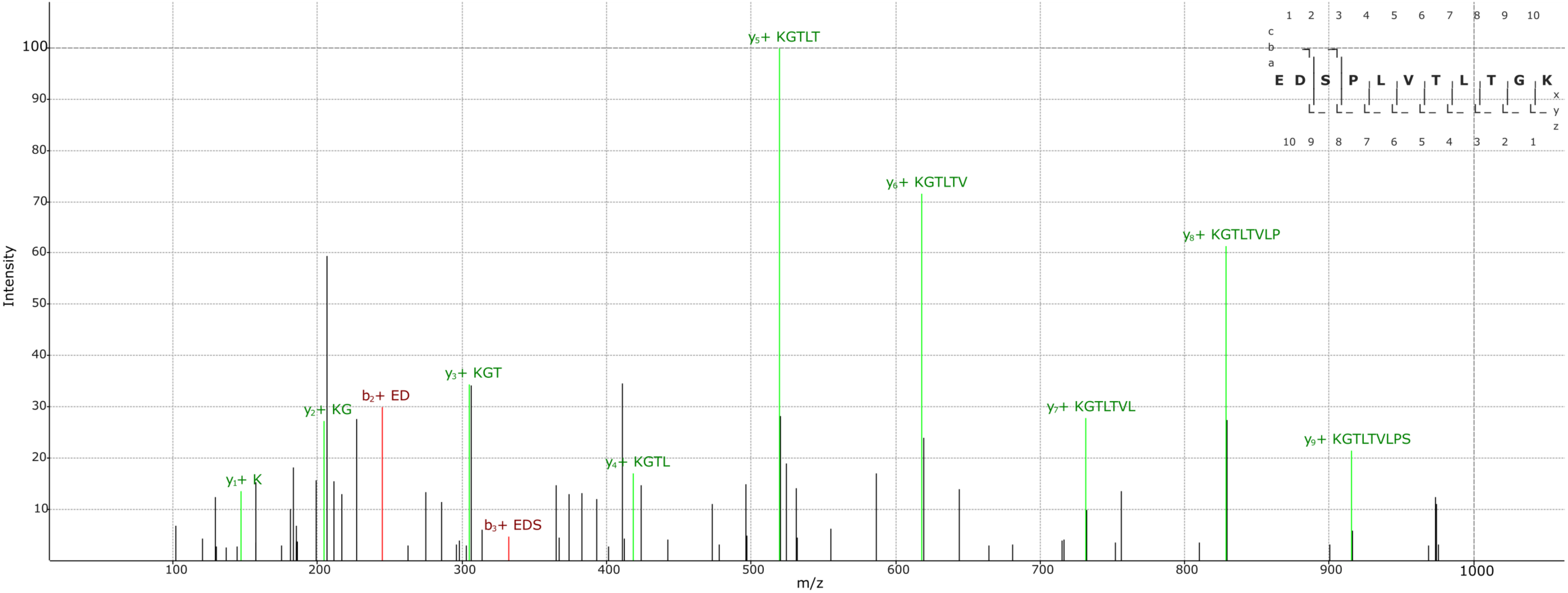

Precursor m/z:	580.3
Peptide charge:	2
Score:	7.14E-14

NSSHWAEEDTILSGVK, N-acetyltransferase YodP

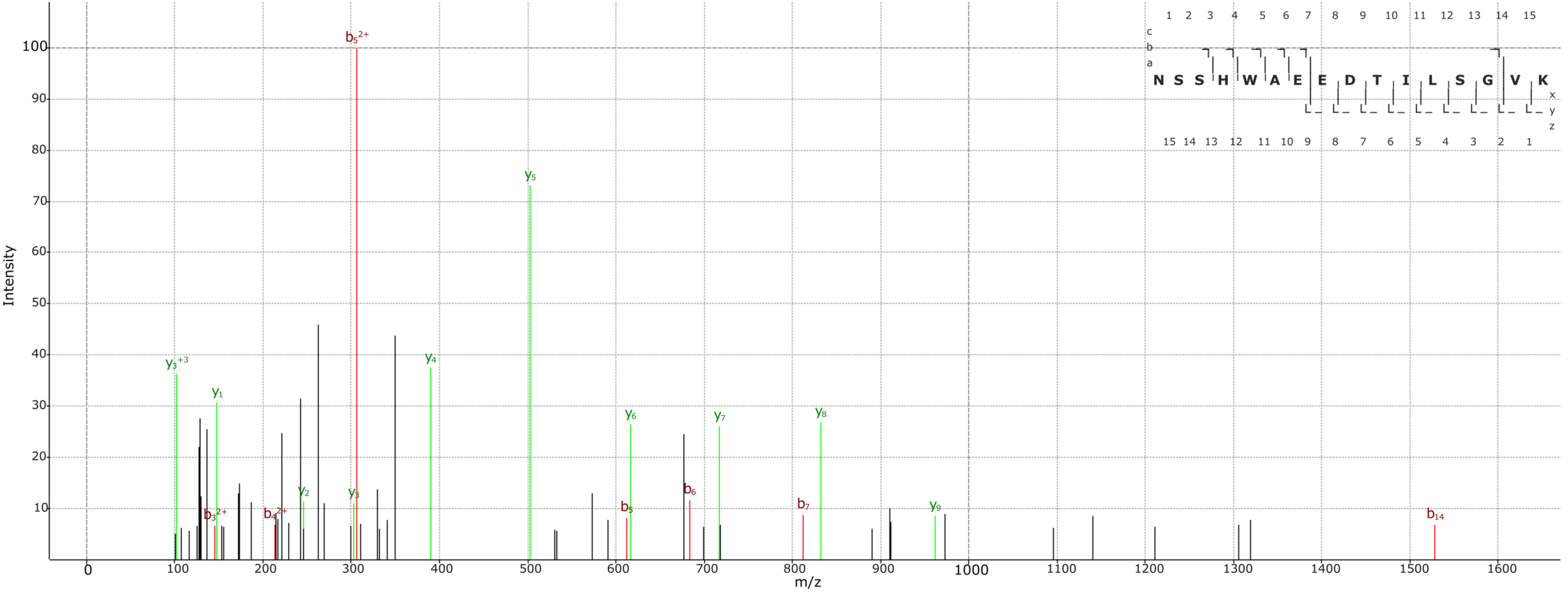

Precursor m/z:	591.6
Peptide charge:	3
Score:	8.03E-14

AQASGASIQSTNASYGTEFATETDVHAVK, Small, acid-soluble spore protein gamma-type

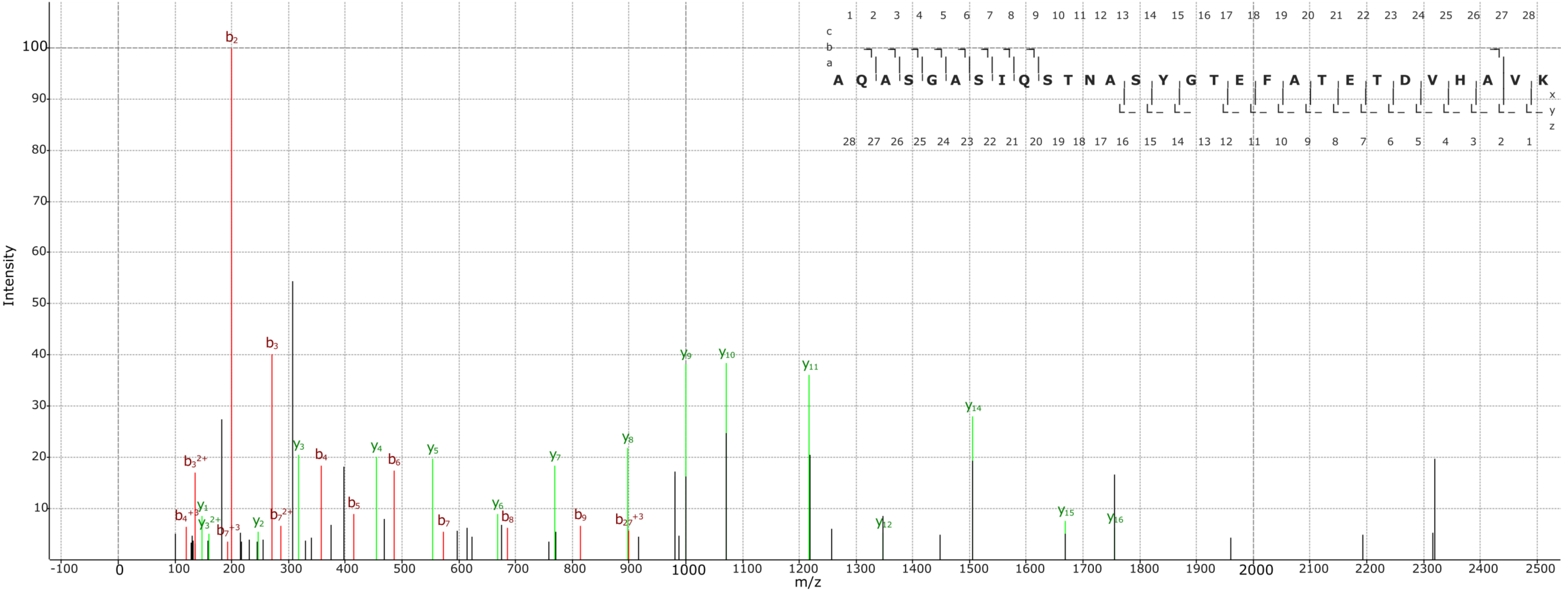

Precursor m/z:	981.5
Peptide charge:	3
Score:	3.43E-27

VLTSGQEAVSHNFLGDK, putative 3-hydroxyacyl-CoA dehydrogenase

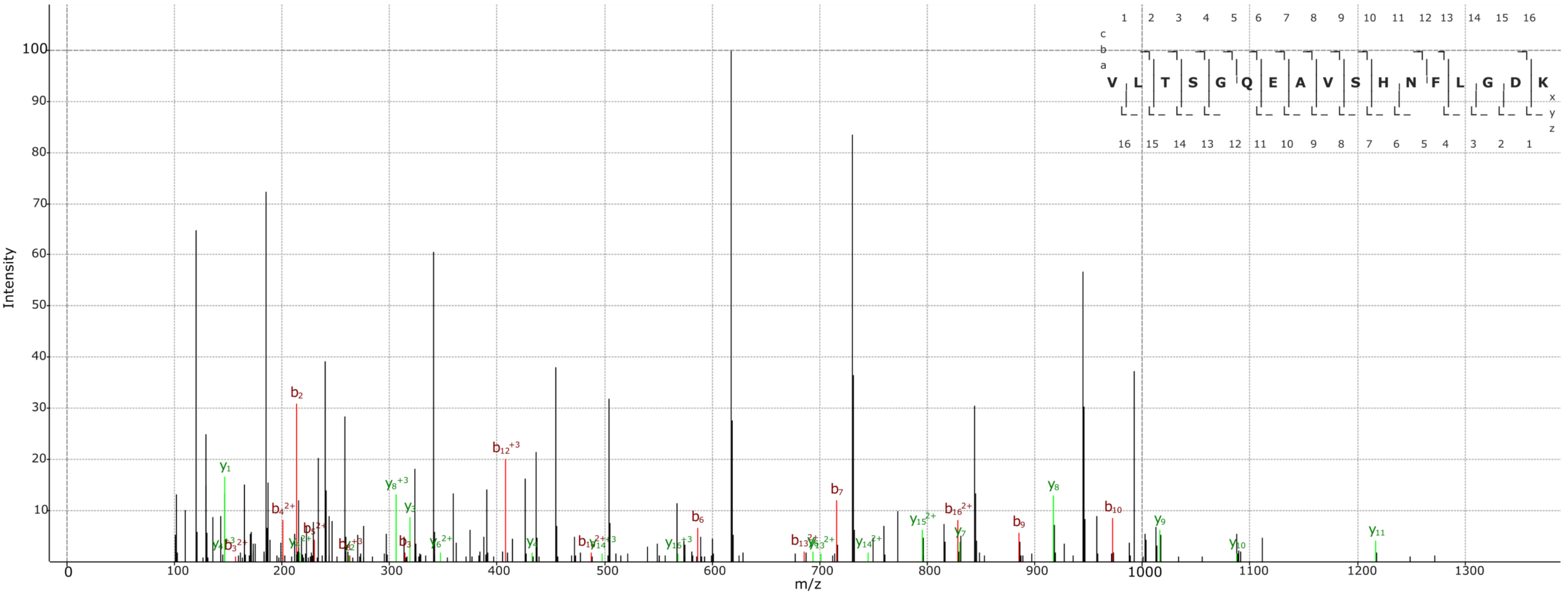

Precursor m/z:	601.6
Peptide charge:	3
Score:	2.42E-10
